# Trends in stroke severity at hospital admission and rehabilitation discharge before and during the COVID-19 pandemic in Hesse, Germany: a register-based study

**DOI:** 10.1186/s42466-024-00308-5

**Published:** 2024-03-07

**Authors:** Matthias Hans Belau, Björn Misselwitz, Uta Meyding-Lamadé, Burc Bassa

**Affiliations:** 1 Institute of Medical Biometry and Epidemiology, University Medical Center Hamburg-Eppendorf, Martinistraße 52, 20246 Hamburg, Germany; 2 Federal State Consortium of Quality Assurance Hesse, Eschborn, Germany; 3 Department of Neurology, Krankenhaus Nordwest, Frankfurt (Main), Germany

**Keywords:** Stroke severity, Hospital admission, Rehabilitation discharge, COVID-19, Germany

## Abstract

**Background:**

The COVID-19 pandemic has affected acute stroke care, resulting in a decrease in stroke admissions worldwide. We examined trends in stroke severity at hospital admission, including (1) probable need for rehabilitation (National Institutes of Health Stroke Scale score > 6 points) and (2) probable need for assistance (modified Rankin Scale score > 2 points), and discharge to rehabilitation after acute care among inpatients with acute ischemic stroke and intracerebral hemorrhage.

**Methods:**

We compared quality assurance data for acute ischemic stroke and intracerebral hemorrhage during the pandemic with the period before the pandemic in Hesse, Germany, using logistic regression analyses.

**Results:**

Fewer inpatients with a probable need for rehabilitation were present at the beginning of the second wave of the COVID-19 pandemic in September 2020 (adjusted OR (aOR) 0.85, 95% CI [0.73, 0.99]), at the end of the second national lockdown in May 2021 (aOR 0.81, 95% CI [0.70, 0.94]), and at the approaching peak of COVID-19 wave 4 in November 2021 (aOR 0.79, 95% CI [0.68, 091]). Rates of probable need for assistance were significantly lower at the beginning of COVID-19 wave 2 in August 2020 (aOR 0.87, 95% CI [0.77, 0.99]) and at the beginning of COVID-19 wave 3 in March 2021 (aOR 0.80, 95% CI [0.71, 0.91]). Rates of discharge to rehabilitation were lower from the beginning in October 2020 to the peak of COVID-19 wave 2 in December 2020 (aOR 0.83, 95% CI [0.77, 0.90]), at the beginning and end of COVID-19 wave 3 in March 2021 and May 2021 (aOR 0.86, 95% CI [0.79, 0.92]), respectively, and at the beginning of COVID-19 wave 4 in October 2021 (aOR 0.86, 95% CI [0.76, 0.98]).

**Conclusions:**

The results suggest that the COVID-19 pandemic had an impact on stroke management during the pandemic, but the absolute difference in stroke severity at hospital admission and discharge to rehabilitation was small.

## Background

Stroke is a leading cause of death and disability in Germany [1], with nearly 140 new cases per 100,000 population annually, including acute ischemic stroke (AIS) and intracerebral hemorrhage (ICH). Rapid stroke detection, acute stroke treatment, and rehabilitation are of paramount importance to reduce morbidity and mortality in these patients [2]. The COVID-19 pandemic has affected all levels of stroke care, resulting in a decrease in stroke admissions [3]. Although the exact reasons for the decline are unknown, physical distancing measures, and psychological factors, including fear of hospital-acquired infections, may have played a role [4].

A few studies showed longer times from last known well to emergency department presentation with stroke-like symptoms during the pandemic [5, 6]. Some studies found no differences in stroke severity or early outcomes [5, 7, 8], while others did [6]. A nationwide cohort study [9] found a sharp decrease in hospitalizations for AIS (− 17.4%) and ICH (− 15.8%) in Germany during the first wave of the pandemic compared with the pre-pandemic period. The rate of intravenous thrombolysis in patients with AIS was comparable (pre-pandemic vs. pandemic: 16.4 vs. 16.6%), whereas the rate of mechanical thrombectomy (7.7 vs. 8.1%) was significantly higher during the pandemic period [9]. In addition, in-hospital mortality was significantly higher in patients with AIS. Further investigation revealed a smaller decrease in hospitalizations for AIS during the second wave of the COVID-19 pandemic compared with the pre-pandemic period [10]. Another study [11] evaluating nationwide data of hospitalized patients with AIS in Germany showed differences in mortality and medical management between AIS patients with and without concomitant SARS-CoV-2 infection. Some studies [12] reported a decrease in acute rehabilitation discharges and another [13] showed no change. There is relatively little information on the impact of the pandemic on acute stroke admissions and discharge to rehabilitation in Germany.

Rehabilitation after stroke is an important part of stroke care [14]. The chances of living independently are significantly higher for stroke patients who receive early and intensive rehabilitation [15]. We used data from the Stroke Inpatient Quality Assurance Registry of the German state of Hesse to examine trends in stroke severity at hospital admission and discharge to rehabilitation in patients with AIS and ICH using registry-based inpatient data. The specific aim of this study was to compare the rates during the pandemic with the period before the pandemic.

## Methods

### Study population and procedure

The mandatory Stroke Inpatient Quality Assurance Registry covers the entire federal state of Hesse, Germany (6.3 million inhabitants). The registry is organized by the State Consortium Quality Assurance Hesse (www.lagqh.de) and represents the entire hospital landscape in Hesse. The completeness of the data was checked by matching the data with the hospitals' billing data. Compulsory documentation includes all patients over the age of 18 who are admitted to the hospital within 7 days after the onset of stroke. Information was collected using a standardized questionnaire.

**Fig. 1:**
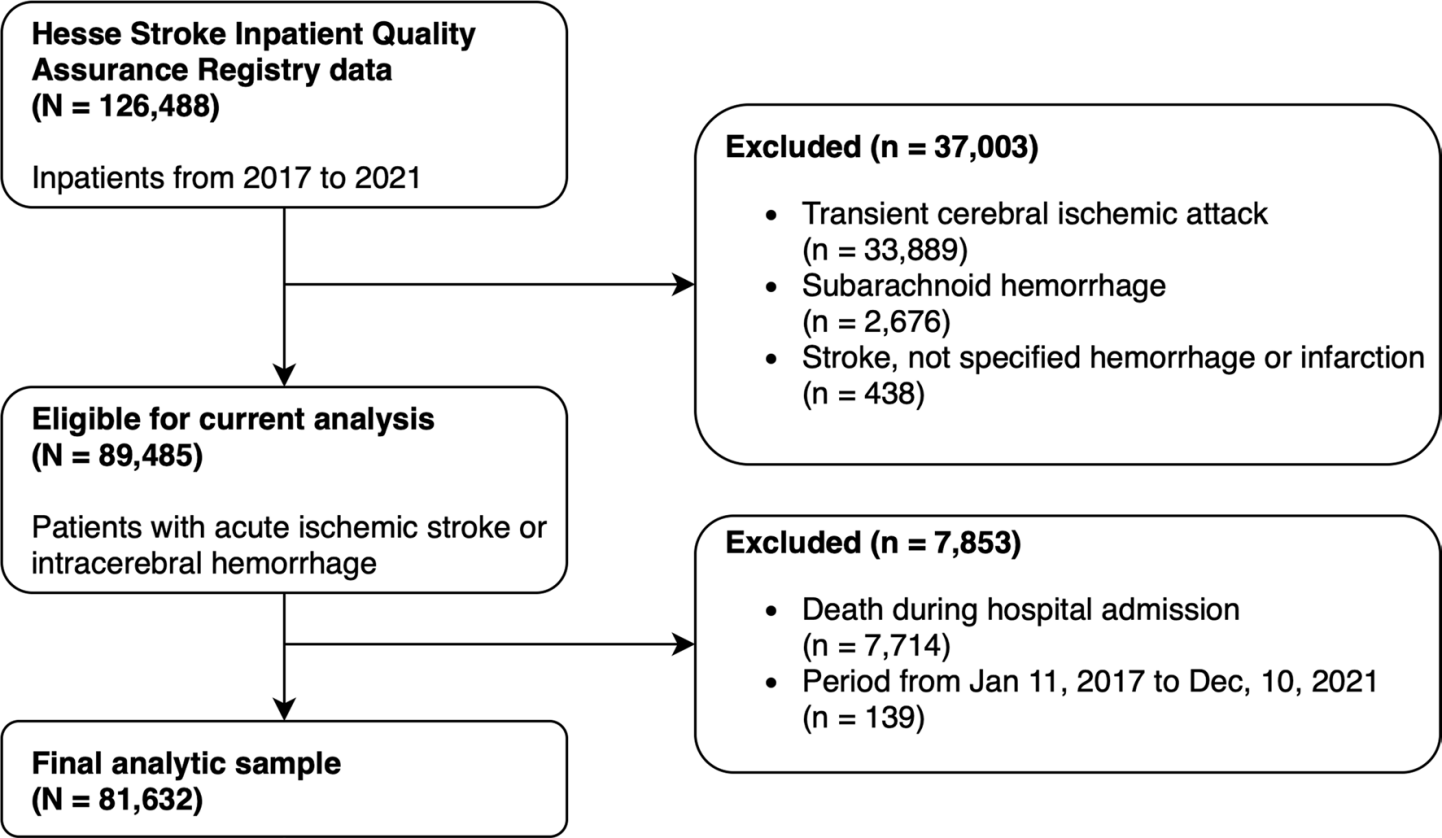
Inpatient Quality Assurance Registry data flow from the total number to the analytical sample for the current analysis

We analyzed data from all inpatients treated in Hesse between 2017 and 2021 with a main diagnosis of AIS (ICD-10 code: I63) or ICH (ICD-10 code: I61). Patients with a main diagnosis of transient cerebral ischemic attack (ICD-10 code: G45), subarachnoid hemorrhage (ICD-10 code: I60), and stroke not specified as hemorrhage or infarction (ICD-10 code: I64) as well as those who died at admission to the hospital were excluded. An overview is shown in Fig. 1.

### Exposure

The exposure of interest was the COVID-19 pandemic. The pandemic was declared by the World Health Organization on March 11, 2020 [16], and we used this date as the cutoff date for comparing the pre-pandemic and pandemic groups. For the comparison of monthly periods before and during the pandemic, we used a truncated period from January 11, 2017, to December 10, 2021, resulting in an analysis sample of N = 81,632 (see Fig. 1).

### COVID-19 infections and nationwide lockdowns

Weekly rates of COVID-19 infections per 100,000 of the general population in Germany, using official data reported under the German Protection against Infection Act [17], were used to show trends in the COVID-19 pandemic. We also show the times of national lockdowns during the COVID-19 pandemic, imposed by the German government to keep the number of new infections as low as possible and prevent the healthcare system from collapsing. The first lockdown went into effect on March 22, 2020, and ended on May 4, 2020. The second lockdown went into effect on November 2, 2020, and varied by region (called "lockdown light"). From January 6, 2021, to May 28, 2021, very strict nationwide rules were again in effect.

### Outcome measures

The outcome measures were (1) stroke severity rate on admission, as assessed by the National Institutes of Health Stroke Scale (NIHSS) as a measure of overall neurological impairment [18] and the modified Rankin Scale (mRS) as a measure of neurological disability [19, 20], and (2) the rate of discharge to rehabilitation.

The NIHSS is a 15-item neurological examination stroke scale used by a trained observer to assess the effects of a stroke on consciousness, language, neglect, visual field loss, extraocular movement, motor strength, ataxia, dysarthria, and sensory loss [18, 21]. Ratings for each item are scored on a 3- to 5-point scale, with 0 being normal and an allowance for untestable items. Scores range from 0 to 42, with higher scores indicating greater severity. We used a score of 6 as the cutoff value because higher scores typically require acute inpatient rehabilitation [22].

The mRS is a 1-item measure that assesses the degree of disability or dependence in daily activities in stroke patients [19, 20]. Scores range from 0 to 5, with higher scores indicating greater disability. A separate category of 6 is usually added for patients who have died. We used a score of 2 as the cutoff because higher scores indicate a probable need for assistance with activities of daily living.

Discharge to rehabilitation includes inpatients with planned rehabilitation after discharge from acute care or direct transfer to a rehabilitation facility. Inpatients with "discharge code 06" (transfer to another hospital), "discharge code 10" (transfer to a nursing facility), and "discharge code 11" (transfer to hospice) were excluded.

### Covariates

Age, sex (female, male), stroke type (AIS, ICH), and mRS score at discharge were included as covariables. Age was categorized into the following 3 age groups (18–49, 50–74, 75 +) due to violation of linearity in logistic regression analyses (see below).

### Statistical analyses

All analyses were conducted using STATA MP in version 18. Descriptive statistics were used to examine the characteristics of the stroke inpatients. The percentage change in stroke severity (NIHSS > 6 points; mRS > 2 points), and discharge to rehabilitation was calculated by subtracting the monthly rate from 2020 to 2021 from the monthly rate before the pandemic (average from 2017 to 2020). In addition, weekly rates of COVID-19 infection in the general population per 100,000 are plotted against monthly stroke severity, and discharge to rehabilitation rates to descriptively show patterns during the COVID-19 pandemic. Multiple logistic regression was applied to compare stroke severity (NIHSS > 6 points; mRS > 2 points), and discharge to rehabilitation in 2020 to 2021 versus the 2017 to 2020 average of the 23 time periods. Adjustments were made for age group (as dummy variables), sex, stroke type, and mRS at discharge (only discharge to rehabilitation analyses). We computed odds ratios (ORs) and 95% confidence intervals (CIs), and the significance level was set at *p* < 0.05.

## Results

From January 11, 2017, to December 10, 2021, n = 73,420 stroke patients with AIS and n = 8212 stroke patients with ICH were treated in hospitals in Hesse. In the years before the pandemic, n = 49,670 patients received acute stroke care in a hospital (2017: n = 15,973; 2018: n = 16,538; 2019: n = 16,699; 2020: n = 460), and during the pandemic, n = 31,962 patients were treated in a hospital (2020: n = 15,684; 2021: n = 16,278).

Table 1 shows the characteristics of the hospitalized stroke patients. The majority were male (53.5%), 75 years and older (54.9%), and diagnosed with AIS (89.9%). The median (Q1, Q3) NIHSS and mRS scores on admission were 3 points (2, 7 points) and 3 points (2, 4 points), respectively. Overall, 45.0% of inpatients were discharged to rehabilitation and 7.9% died during hospitalization. Of the inpatients transferred to rehabilitation, 17.9% were transferred directly (pre-pandemic vs pandemic: 18.3 vs. 16.7%), 32.7% were initially discharged home (pre-pandemic vs pandemic: 32.8 vs. 32.6%) and 1.2% were initially discharged to a nursing facility (pre-pandemic vs. pandemic: 1.2 vs. 1.2%).

**Table 1:** Characteristics of stroke patients in Hesse, Germany, between January 11, 2017, and December 10, 2021

	Cases, n (%)		Total N = 81,632
	Pre-pandemic, January 11, 2017—March 10, 2020 n = 49,670	During pandemic, March 11, 2020—December 10, 2021 n = 31,962	
Age			
Mean (SD)	73.5 (12.9)	73.5 (12.9)	73.5 (12.9)
Median	76	76	76
Q1, Q3	66, 83	65, 83	66, 83
Min, Max	18, 106	18, 105	18, 106
Age group			
18–49	2,370 (4.8)	1,488 (4.6)	3,858 (4.7)
50–74	19,770 (39.8)	13,154 (41.2)	32,924 (40.4)
75 +	27,530 (55.4)	17,320 (54.2)	44,850 (54.9)
Sex			
Female	23,174 (46.6)	14,733 (46.1)	37,907 (46.4)
Male	26,483 (53.3)	17,229 (53.9)	43,712 (53.5)
*Missing values*	13 (0.1)	0 (0.0)	13 (0.1)
Stroke type			
Hemorrhagic	4999 (10.1)	3213 (10.1)	8212 (10.1)
Ischemic	44,671 (89.9)	28,749 (89.9)	73,420 (89.9)
Admission NIHSS score			
Mean (SD)	5.6 (6.1)	5.5 (6.2)	5.6 (6.1)
≤ 6 points	31,765 (63.9)	20,645 (64.6)	52,410 (64.2)
> 6 points	12,844 (25.9)	8056 (25.2)	20,900 (25.6)
*Missing values*	5061 (10.2)	3261 (10.2)	8322 (10.2)
Admission mRS score			
Mean (SD)	2.8 (1.4)	2.8 (1.5)	2.8 (1.4)
≤ 2 points	20,257 (40.8)	13,439 (42.0)	33,696 (41.3)
> 2 points	29,413 (59.2)	18,523 (58.0)	47,936 (58.7)
Discharge mRS score			
Mean (SD)	2.4 (1.8)	2.4 (1.8)	2.4 (1.8)
≤ 2 points	27,809 (55.9)	18,129 (56.7)	45,938 (56.3)
> 2 points	16,414 (33.1)	10,431 (32.6)	26,845 (32.9)
Deceased	3878 (7.8)	2622 (8.2)	6500 (7.9)
*Missing values*	1569 (3.2)	780 (2.4)	2349 (2.9)
Discharge to rehabilitation			
No	21,610 (43.5)	14,451 (45.2)	36,061 (44.2)
Yes	22,613 (45.5)	14,109 (44.2)	36,722 (45.0)
Deceased	3878 (7.8)	2622 (8.2)	6500 (7.9)
*Missing values*	1569 (3.2)	780 (2.4)	2349 (2.9)

n, quantity; %, proportion; *SD* standard deviation, *Q1* first Quintile, *Q3* third quintile

**Fig. 2:**
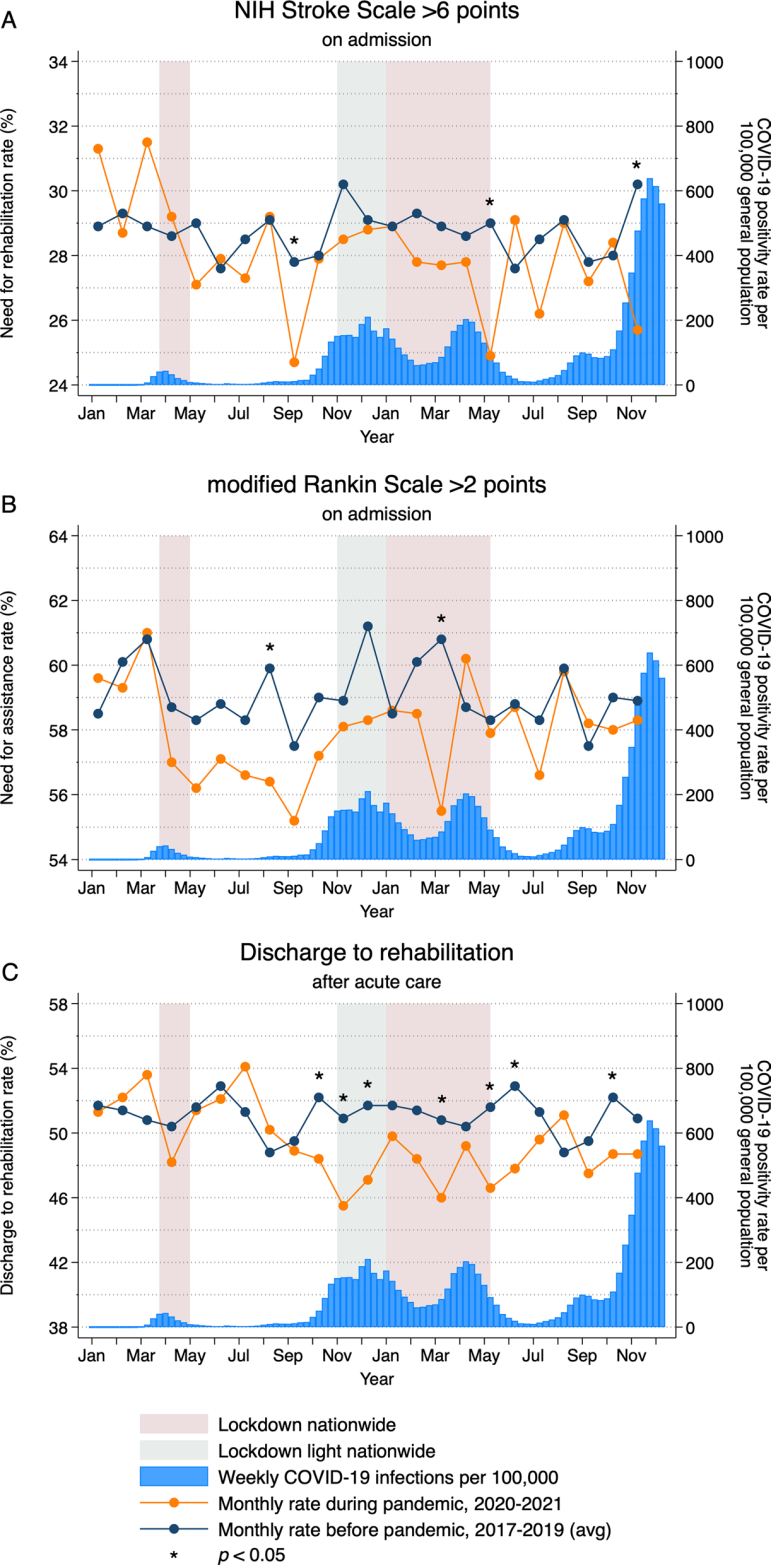
Weekly COVID-19 infection rates in the general population and monthly **A** need for rehabilitation (NIHSS score >6 points) and **B** need for assistance (mRS score >2 points) at hospital admission and **C** discharge to rehabilitation rates in Hesse, Germany, between January 11, 2017 and December 10, 2021

Figure 2 displays weekly rates of COVID-19 infection in the general population and monthly rates of (A) probable need for rehabilitation (NIHSS score > 6 points) and (B) probable need for assistance (mRS score > 2 points) at hospital admission, and (C) discharge to rehabilitation. The figures display four COVID-19 waves with peaks in April 2020, December 2020, April 2021, and December 2021.

Figure 2A shows that the rate of NIHSS score > 6 points at hospital admission was above the 2017–2020 average estimates in January and March 2020, and then remained below the 2017–2020 average estimates for the remainder of the study period (except in June 2021). Figure 2B displays a mostly similar pattern for the rate of mRS score > 2 points at admission, with estimates during the pandemic below the 2017–2020 average estimates for the remainder of the study period (except in April 2021). Figure 2C shows that the rate of discharges to rehabilitation increased above 2017–2020 average estimates from January to March 2020, decreased during the first national lockdown and remained below 2017–2020 average estimates in May and June, were above 2017–2020 average estimates again in July and August 2020, and then remained below 2017–2020 average estimates for the remainder of the study period (except in August 2021).

Table 2 displays the statistical estimates comparing 2020 to 2021 with the average of 2017 to 2020. For NIHSS > 6 points, rates were significantly lower than pre-pandemic levels from September 11 to October 10, 2020 (Change = − 3.1%; adjusted OR (aOR) 0.85, 95% CI [0.73, 0.99]), May 11 to June 10, 2021 (Change = − 4.1%; aOR 0.81, 95% CI [0.70, 0.94]), and November 11 to December 10, 2021 (Change = − 4.5; aOR 0.79, 95% CI [0.68, 091]). For mRS > 2 points, rates were significantly lower than pre-pandemic levels from August 11 to September 10, 2020 (Change = − 3.5; aOR 0.87, 95% CI [0.77, 0.99]), and March 11 to April 10, 2021 (Change = − 5.3; aOR 0.80, 95% CI [0.71, 0.91]). For discharge to rehabilitation, rates were significantly lower than pre-pandemic levels from October 11 to November 10, 2020 (Change = − 3.8; aOR 0.86, 95% CI [0.75, 0.98]), November 11 to December 10, 2020 (Change = − 5.4; aOR 0.80, 95% CI [0.70, 0.92]), December 11, 2020, to January 10, 2021 (Change = − 4.0; aOR 0.82, 95% CI [0.72, 0.95]), March 11 to April 10, 2021 (Change = − 4.8; aOR 0.82, 95% CI [0.72, 0.93]), May 11 to June 10, 2021 (Change = − 5.0; aOR 0.82, 95% CI [0.72, 0.93]), June 11 to July 10, 2021 (Change − 5.1; aOR 0.80, 95% CI [0.70, 0.91]), and October 11 to November 10, 2021 (Change = − 3.5; aOR 0.86, 95% CI [0.76, 0.98]).

**Table 2:** Association between stroke severity on admission and discharge to rehabilitation comparing COVID-19 pandemic period 2020 to 2021 with pre-pandemic period 2017 to 2020

Monthly time period	Year	NIH Stroke Scale on admission					Modified Rankin Scale on admission					Discharge to rehabilitation after acute care				
		NIHSS > 6 points, %			Estimates		mRS > 2 points, %			Estimates		Rehabilitation rate, %			Estimates	
		Before pandemic^a^	During pandemic	Change, %	Crude OR (95% CI)	Adjusted^b^ OR (95% CI)	Before pandemic^a^	During pandemic	Change, %	Crude OR (95% CI)	Adjusted^b^ OR (95% CI)	Before pandemic^a^	During pandemic	Change, %	Crude OR (95% CI)	Adjusted ^c^ OR (95% CI)
Jan 11 to Feb10	2020 vs 2017–2019	28.9	31.3	2.4	1.12 (0.97, 1.28)	1.11 (0.97, 1.28)	58.5	59.6	1.1	1.04 (0.92, 1.18)	1.03 (0.91, 1.17)	51.7	51.3	− 0.4	0.98 (0.86, 1.11)	0.98 (0.86, 1.11)
Feb 11 to Mar 10	2020 vs 2017–2019	29.3	28.7	− 0.6	0.97 (0.84, 1.11)	0.97 (0.84, 1.12)	60.1	59.3	− 0.8	0.96 (0.85, 1.09)	0.96 (0.85, 1.09)	51.4	52.2	0.8	1.03 (0.90, 1.17)	1.02 (0.89, 1.16)
Mar 11 to Apr 10	2020 vs 2017–2019	28.9	31.5	2.6	1.13 (0.98, 1.29)	1.11 (0.97, 1.28)	60.8	61.0	0.2	1.00 (0.88, 1.13)	0.99 (0.88, 1.13)	50.8	53.6	2.8	1.11 (0.98, 1.27)	1.11 (0.97, 1.26)
Apr 11 to May 10	2020 vs 2017–2019	28.6	29.2	− 0.6	1.02 (0.88, 1.19)	1.04 (0.89, 1.21)	58.7	57.0	− 1.7	0.93 (0.81, 1.06)	0.91 (0.79, 1.04)	50.4	48.2	− 2.2	0.91 (0.79, 1.04)	0.90 (0.78, 1.03)
May 11 to Jun 10	2020 vs 2017–2019	29.0	27.1	− 1.9	0.91 (0.79, 1.05)	0.90 (0.78, 1.04)	58.3	56.2	− 2.1	0.92 (0.81, 1.03)	0.90 (0.80, 1.02)	51.6	51.4	− 0.2	0.99 (0.87, 1.12)	1.00 (0.87, 1.13)
Jun 11 to Jul 10	2020 vs 2017–2019	27.6	27.9	0.3	1.01 (0.87, 1.17)	1.03 (0.89, 1.19)	58.8	57.1	− 1.7	0.93 (0.82, 1.06)	0.94 (0.83, 1.07)	52.9	52.1	0.8	0.96 (0.84, 1.10)	0.95 (0.83, 1.09)
Jul 11 to Aug 10	2020 vs 2017–2019	28.5	27.3	− 1.2	0.94 (0.81, 1.08)	0.93 (0.81, 1.08)	58.3	56.6	− 1.7	0.93 (0.81, 1.05)	0.93 (0.82, 1.06)	51.3	54.1	2.8	1.12 (0.98, 1.28)	1.13 (0.99, 1.29)
Aug 11 to Sep 10	2020 vs 2017–2019	29.1	29.2	0.1	1.00 (0.87, 1.15)	1.04 (0.90, 1.20)	59.9	56.4	− 3.5	0.86 (0.76, 0.97)	0.87 (0.77, 0.99)	48.8	50.2	1.4	1.05 (0.92, 1.20)	1.04 (0.91, 1.18)
Sep 11 to Oct 10	2020 vs 2017–2019	27.8	24.7	− 3.1	0.85 (0.73, 0.99)	0.85 (0.73, 0.99)	57.5	55.2	− 2.3	0.91 (0.80, 1.03)	0.93 (0.81, 1.06)	49.5	48.9	− 0.6	0.97 (0.85, 1.11)	0.97 (0.85, 1.11)
Oct 11 to Nov 10	2020 vs 2017–2019	28.0	27.9	− 0.1	0.99 (0.86, 1.15)	0.98 (0.85, 1.14)	59.0	57.2	− 1.8	0.92 (0.81, 1.04)	0.92 (0.81, 1.04)	52.2	48.4	− 3.8	0.85 (0.75, 0.97)	0.86 (0.75, 0.98)
Nov 11 to Dec 10	2020 vs 2017–2019	30.2	28.5	− 1.7	0.92 (0.79, 1.06)	0.91 (0.79, 1.06)	58.9	58.1	− 0.8	0.96 (0.85, 1.09)	0.96 (0.84, 1.09)	50.9	45.5	− 5.4	0.80 (0.70, 0.91)	0.80 (0.70, 0.92)
Dec 11 to Jan 10	2020–2021 vs 2017–2019	29.1	28.8	− 0.3	0.98 (0.85, 1.14)	1.01 (0.86, 1.17)	61.2	58.3	− 2.9	0.88 (0.77, 1.00)	0.91 (0.79, 1.04)	51.7	47.1	− 4.0	0.83 (0.72, 0.95)	0.82 (0.72, 0.95)
Jan 11 to Feb 10	2021 vs 2017–2019	28.9	28.9	0.0	1.00 (0.87, 1.15)	0.99 (0.96, 1.14)	58.5	58.6	0.1	1.00 (0.88, 1.13)	1.00 (0.88, 1.13)	51.7	49.8	− 1.9	0.92 (0.81, 1.05)	0.92 (0.81, 1.05)
Feb 11 to Mar 10	2021 vs 2017–2019	29.3	27.8	− 1.5	0.92 (0.79, 1.07)	0.93 (0.80, 1.08)	60.1	58.5	− 1.6	0.93 (0.82, 1.06)	0.94 (0.82, 1.07)	51.4	48.4	− 3.0	0.88 (0.77, 1.01)	0.88 (0.76, 1.00)
Mar 11 to Apr 10	2021 vs 2017–2019	28.9	27.7	− 1.2	0.94 (0.81, 1.08)	0.95 (0.82, 1.10)	60.8	55.5	− 5.3	0.80 (0.70, 0.90)	0.80 (0.71, 0.91)	50.8	46.0	− 4.8	0.82 (0.72, 0.93)	0.82 (0.72, 0.93)
Apr 11 to May 10	2021 vs 2017–2019	28.6	27.8	− 0.8	0.95 (0.82, 1.10)	0.96 (0.83, 1.12)	58.7	60.2	1.5	1.06 (0.93, 1.20)	1.04 (0.91, 1.19)	50.4	49.2	− 1.2	0.95 (0.83, 1.08)	0.94 (0.83, 1.08)
May 11 to Jun 10	2021 vs 2017–2019	29.0	24.9	− 4.1	0.81 (0.70, 0.93)	0.81 (0.70, 0.94)	58.3	57.9	− 0.4	0.98 (0.83, 1.10)	0.98 (0.86, 1.10)	51.6	46.6	− 5.0	0.82 (0.72, 0.929	0.82 (0.72, 0.93)
Jun 11 to Jul 10	2021 vs 2017–2019	27.6	29.1	1.5	1.08 (0.93, 1.25)	1.09 (0.95, 1.27)	58.8	58.7	− 0.1	0.99 (0.87, 1.13)	1.00 (0.88, 1.14)	52.9	47.8	− 5.1	0.81 (0.71, 0.93)	0.80 (0.70, 0.91)
Jul 11 to Aug 10	2021 vs 2017–2019	28.5	26.2	− 2.3	0.89 (0.76, 1.03)	0.89 (0.77, 1.04)	58.3	56.6	− 1.7	0.93 (0.82, 1.05)	0.93 (0.82, 1.06)	51.3	49.6	− 1.7	0.93 (0.81, 1.06)	0.93 (0.81, 1.06)
Aug 11 to Sep 10	2021 vs 2017–2019	29.1	29.0	− 0.1	0.99 (0.86, 1.14)	0.98 (0.85, 1.13)	59.9	59.8	− 0.1	0.99 (0.88, 1.12)	0.97 (0.85, 1.10)	48.8	51.1	1.3	1.09 (0.96, 1.24)	1.09 (0.96, 1.24)
Sep 11 to Oct 10	2021 vs 2017–2019	27.8	27.2	− 0.6	0.97 (0.83, 1.12)	0.99 (0.85, 1.15)	57.5	58.2	0.7	1.03 (0.90, 1.17)	1.07 (0.94, 1.22)	49.5	47.5	− 2.0	0.92 (0.80, 1.05)	0.91 (0.79, 1.04)
Oct 11 to Nov 10	2021 vs 2017–2019	28.0	28.4	0.4	1.01 (0.88, 1.17)	1.03 (0.89, 1.19)	59.0	58.0	− 1.0	0.95 (0.84, 1.08)	0.99 (0.88, 1.13)	52.2	48.7	− 3.5	0.87 (0.76, 0.98)	0.86 (0.76, 0.98)
Nov 11 to Dec 10	2021 vs 2017–2019	30.2	25.7	− 4.5	0.80 (0.69, 0.92)	0.79 (0.68, 0.91)	58.9	58.3	− 0.6	0.97 (0.86, 1.10)	0.96 (85, 1.09)	50.9	48.7	− 2.2	0.91 (0.80, 1.03)	0.91 (0.80, 1.04)

%, proportion, *OR* odds ratio, *CI* confidence interval

^a^Average from 2017 to 2019

^b^Adjusted for age, sex, and type of stroke

^c^Adjusted for age, sex, type of stroke, and mRS score at discharge

## Discussion

This study aimed to examine trends in stroke severity at hospital admission and discharge to rehabilitation after acute care among inpatients with AIS and ICH and to compare rates during the pandemic with the period before the pandemic. With the onset of the pandemic, there was a slight decrease in the absolute number of stroke patients treated in hospitals, which is consistent with another study [9] and may be a direct result of the implementation of stay-at-home orders and lockdowns enforced by local and federal governments [23]. Overall, however, there was no significant reduction in the number of cases during the pandemic compared with the years before the pandemic.

Furthermore, the results show that there were significantly fewer inpatients requiring rehabilitation due to neurological deficits, as indicated by an NIHSS > 6, at the beginning of the second wave of the COVID-19 pandemic in September 2020, as well as at the end of the second national lockdown in May 2021 and the approaching peak of COVID-19 wave 4 in November 2021. The results for the probable need for assistance due to major neurological deficits, as indicated by mRS > 2 points, show significantly lower rates at the beginning of COVID-19 wave 2 in August 2020 and at the beginning of COVID-19 wave 3 in March 2021. Some studies found no differences in NIHSS and mRS at admission [5, 7], while others found differences [6, 24, 25], but in contrast to our results, patients had higher presenting NIHSS and mRS during the pandemic. However, the periods considered in these studies were short and involved small numbers of cases.

The results for discharge to rehabilitation show lower rates from the beginning in October 2020 to the peak of COVID-19 wave 2 in December 2020, at the beginning and end of COVID-19 wave 3 in March 2021 and May 2021, respectively, and at the beginning of COVID-19 wave 4 in October 2021. In a study of 507 stroke inpatients admitted to a comprehensive stroke center in New Jersey, USA, 6 months before (10/2019–03/2020) and during (04/2020–09/2020) the COVID-19 pandemic, the odds of being discharged to rehabilitation were significantly lower during the COVID-19 pandemic [12]. The lowest rate of discharge to rehabilitation occurred between March and May 2020, the beginning and end of the first wave of COVID-19 in New Jersey [26], which is somewhat consistent with our findings. Another study of 1,160 inpatients with AIS in Tyrol, Austria, showed that post-stroke care in rehabilitation centers decreased in 2020 compared with pre-pandemic years [8]. In a survey of 426 stroke care providers from 55 countries, rehabilitation was identified as the area of stroke care most affected by the pandemic [27]. The lack of resources to ensure bed availability for all patients during the pandemic was only one of the issues that may have led to the decline in discharge rates to rehabilitation during the pandemic, and the exact causes need to be further investigated.

The major strength of this study is its large sample size based on mandatory quality assurance registry data of all hospitalized AIS and ICH patients in Hesse, Germany. Limitations were that we could not account for comorbidities, COVID-19 infections in patients, and other relevant measures such as the Barthel Index, which systematically measures basic activities of daily living. Immediately after the stroke, at the time of admission, the mRS should be interpreted with caution and the Barthel Index is more reliable [28]. However, the mRS helps discriminate between patients who need assistance with activities of daily living and those who do not. Another potential confounding factor that we were unable to adjust for is ethnicity [6, 12] and should be considered in future studies. Further limitations include the number of missing values in the NIHSS is more than 10%, which may lead to estimation bias [29]. Although the proportion of missing values is the same in both groups (pre-pandemic, during the pandemic), it is not possible to infer the missing mechanism from the secondary data. Finally, it is important to note that our data only include stroke patients treated in a hospital in Hesse between 2017 and 2021 with a primary diagnosis of AIS or ICH, and the results cannot be generalized to all stroke patients in Hesse and Germany. As these data are not available for the whole of Germany, large-scale analyses are very difficult to perform.

Despite these limitations, this study contributes to a better understanding of the relationship between stroke severity on admission and discharge to rehabilitation, comparing the COVID-19 pandemic period 2020 to 2021 with the pre-pandemic period 2017 to 2020. Future research should continue to explore this relationship to better prepare for a future pandemic in the spirit of the lessons learned from the COVID-19 pandemic.

## Conclusions

First, our results suggest that the absolute number of stroke patients treated in hospitals decreased with the onset of the COVID-19 pandemic. Second, our results reveal that the probable need for rehabilitation and assistance with activities of daily living after acute stroke during the pandemic does not differ significantly from pre-pandemic years, with a few exceptions. Third, planned rehabilitation after discharge from acute care or direct transfer to a rehabilitation facility differs significantly from the pre-pandemic years, especially during the second national lockdown in the second and third COVID-19 waves. Thus, our results suggest that the COVID-19 pandemic had an impact on stroke management during the pandemic, but the absolute difference in stroke severity at hospital admission and discharge to rehabilitation was small.

## Data Availability

The datasets used and/or analyzed during the current study are available from the corresponding author on reasonable request.

## Abbreviations

AIS: Acute ischemic stroke
CI: Confidence interval
ICH: Intracerebral hemorrhage
ICD-10-GM: International statistical classification of diseases, 10th revision, German modification
mRS: Modified rankin scale
NIHSS: National Institutes of Health Stroke Scale
OR: Odds ratio
aOR: Adjusted odds ratio
Q1: Quintile 1
Q3: Quintile 3
SARS-CoV-2: Severe acute respiratory syndrome coronavirus type 2
